# Evaluation of 1cp-LSD for Enhancing Welfare in Shelter Dogs: A Randomized Blind Trial with Ethological Intervention

**DOI:** 10.3390/vetsci13010096

**Published:** 2026-01-19

**Authors:** Elisa Hernández-Álvarez, Cristina Canino-Quijada, Sira Roiz, Octavio P. Luzardo, Luis Alberto Henríquez-Hernández

**Affiliations:** 1 Unit of Toxicology, Clinical Sciences Department, Universidad de Las Palmas de Gran Canaria, Paseo Blas Cabrera Felipe s/n, 35016 Las Palmas de Gran Canaria, Spain; octavio.perez@ulpgc.es; 2 Universidad Fernando Pessoa Canarias, 35450 Guía, Spain; 3 Asociación Científica Psicodélica, 35412 Arucas, Spain; 4 Albergue Insular de Animales, GESPLAN—Cabildo de Gran Canaria, 35415 Arucas, Spain; criscanqui@gmail.com (C.C.-Q.); sroimar@gesplan.es (S.R.); 5 Asociación por el Respeto y el Compromiso con los Animales y la Naturaleza (ARYCAN), 35217 Valsequillo de Gran Canaria, Spain

**Keywords:** animal welfare, shelter dogs, animal behavior, psychedelics, lysergic acid diethylamide, 1-cyclopropionyl lysergic acid diethylamide, ethology, ethological intervention

## Abstract

Shelter environments frequently expose dogs to chronic stress and anxiety, which can compromise their welfare and reduce their chances of adoption. Recent interest in psychedelic-assisted approaches has suggested potential therapeutic applications in veterinary behavioral medicine, although empirical evidence remains scarce. This study aimed to evaluate the combined effects of low-dose 1-cyclopropionyl lysergic acid diethylamide (1cp-LSD), a legal lysergamide prodrug of LSD in several countries, and ethological intervention (EI) on the behavior and welfare of shelter dogs. Twenty dogs were randomly assigned to four groups: pharmacological intervention, ethological intervention, combined treatment, or control. The ethological sessions were conducted by veterinary behaviorists, and pharmacological treatment consisted of 10 µg of 1cp-LSD administered orally for three weeks. Blinded evaluators assessed animals using validated anxiety and welfare scales, including a treatment expectation scale, before, during and after the intervention. Results showed that the combined condition consistently outperformed single interventions, significantly enhancing sociability, calmness, and positive emotional reactivity. Importantly, these improvements persisted for three weeks following treatment cessation, indicating sustained benefits beyond the active intervention phase. These findings provide preliminary evidence for the potential of integrating low doses of psychedelics with behavioral therapy in shelter settings. Future studies with larger cohorts and refined pharmacokinetic data are required to confirm safety, elucidate mechanisms, and optimize protocols for clinical application in veterinary practice.

## Simple Summary

Dogs living in animal shelters are often exposed to chronic stress, which can negatively affect their emotional well-being and reduce their chances of adoption. While behavioral interventions are commonly used to improve welfare in these settings, their effects are often limited in duration. In this study, we explored whether combining a structured ethological intervention with low, non-hallucinogenic doses of 1cp-LSD, a legal LSD analog compound, could produce more sustained improvements in shelter dogs. Twenty dogs were assigned to receive behavioral intervention, pharmacological treatment, both combined, or no intervention. Dogs receiving the combined approach showed greater improvements in calmness, sociability, and positive emotional responses compared to single interventions or controls. Importantly, these benefits persisted several weeks after the treatment ended. Although preliminary, these findings suggest that integrating low-dose psychedelic pharmacology with behavioral support may represent a promising new strategy to enhance welfare in shelter dogs. Further research is needed to confirm safety, understand mechanisms, and determine how such approaches could be ethically applied in veterinary practice.

## 1. Introduction

Spain is among the European countries with the highest rates of companion animal abandonment [1,2]. In March 2025, the Ministry of Social Rights, Consumer Affairs and the 2030 Agenda released the first official nationwide report on animal protection management [3]. This investigation, conducted by the Directorate-General for Animal Rights, constitutes the initial step toward the development of a State Action Plan against Animal Abandonment. The national report was based on surveys conducted across 500 municipalities and 250 animal protection entities, compiling data from the year 2023. A key finding was that more than 80% of dogs and cats admitted to collection centers were not microchipped, specifically 70% of dogs, thereby preventing identification of their owners. A total of 18,764 dogs entered the facilities, with 18,009 leaving, resulting in a retention rate of approximately 4% [3]. These figures, however, refer only to new admissions within that year and do not consider animals that were already housed in the facilities at the beginning of the reporting period. Therefore, the shelter population turnover rate of dogs in shelters is likely to be higher than indicated by the annual admission–discharge balance. According to data from the Affinity Foundation, a total of 170,712 dogs were taken in by animal protection organizations in 2023, increasing to 173,867 in 2024. Of these, 4% were returned to their owners, 49% were adopted, 17% died, and 30% remained unadopted [4]. The typical profile of abandoned dogs housed in shelters corresponds to adult, large-sized, mixed-breed animals, with 77% being adults and 51% crossbred [1,3]. In light of these findings, specific measures are currently under development to reduce abandonment rates and shorten the duration of shelter stays [3]. It is noteworthy that 10% of animal abandonments are attributed to behavioral problems, which, together with the end of the hunting season, represents the fourth most common reason for relinquishment [4].

Behavioral and anxiety disorders are common in domestic dogs, posing substantial challenges for animal welfare, owner–pet relationships, and, in some cases, public health. Among these conditions, noise sensitivity is the most prevalent, reported in 32–39% of cases, followed by general fearfulness (26–29%) and separation anxiety (14–22%) [5,6]. Large epidemiological studies further indicate that up to 85.9% of dogs demonstrate moderate to severe separation- or attachment-related behaviors, while nearly half show signs of fear and anxiety [7]. Several environmental influences—such as insufficient early socialization, inadequate maternal care, and reduced access to daily physical activity—have been linked to increased rates of anxiety disorders in this species [8]. Shelter environments pose considerable challenges to canine welfare, with anxiety and stress representing some of the most critical issues [9]. Anxiety- and fear-related conditions are regarded as major welfare concerns in shelter dogs, often comparable in impact to depressive-like states [10]. Stressors such as high noise levels, overcrowding, limited enrichment, and irregular daily routines have been shown to exacerbate anxiety, as demonstrated by both behavioral indicators and physiological responses, including elevated cortisol concentrations [9,11,12]. Clinical signs commonly associated with shelter-related anxiety include hypervigilance, trembling, hypersalivation, and impaired resting ability [5,9]. Behavioral tools such as the Qualitative Behavioral Assessment consistently highlight descriptors such as “anxious,” “nervous,” and “stressed,” which frequently co-occur with fear and depression-like patterns [9,13]. Nonetheless, anxiety severity is highly variable across individuals, depending on factors such as duration of shelter stay, prior life history, temperament, and the quality of shelter management [9,14]. While some dogs exhibit only moderate physiological activation, others—particularly those with poor early socialization or prolonged stays—demonstrate severe and persistent anxiety, making adaptation and recovery especially difficult [9,15].

Admission to a shelter is a major precipitating factor for behavioral and welfare problems in dogs [10]. In this environment, various strategies are employed to mitigate these negative affective states [16]. Current practices often include the use of psychoactive medications, with trazodone and gabapentin reported as the most commonly administered drugs for managing fear and anxiety in dogs [17]. Reported data indicate that these treatments are commonly administered and are perceived as effective in alleviating anxious behaviors [18]. Alongside pharmacological approaches, non-medication alternatives are also commonly implemented. Despite their widespread use, significant barriers remain, particularly related to treatment costs and uncertainty about therapeutic efficacy [18]. Non-pharmacological interventions have been investigated to mitigate stress in shelter dogs [10]. Human interaction in calm settings can buffer glucocorticoid responses and lower anxiety or aggression, while sensory enrichment—such as music, lavender, or dog-appeasing pheromones—has been linked to reduced arousal, more resting behavior, and less vocalization [14,19]. Although these non-invasive measures underscore the potential of low-cost interventions to improve welfare during shelter confinement, overcrowding, excessive workload, and limited staffing prevent these interventions from being implemented effectively.

Recently, new treatments have been tested to improve the welfare of shelter animals. Specifically, a double-blind trial with cannabidiol (CBD) observed a significant increase in hair cortisol levels, without finding notable differences in behavioral responses to various stimuli, highlighting the importance of considering individual variability in treatment efficacy [20]. Classical psychedelics, including psilocybin, lysergic acid diethylamide (LSD), mescaline, and dimethyltryptamine (DMT), have demonstrated beneficial effects in conditions such as anxiety, depression, and fear of death [21,22,23]. These compounds exert their pharmacological action primarily through the serotonin 5-HT2A receptor [24], while also modulating the default mode network (DMN) [25]. The serotonergic system is conserved in dogs [26], and there is emerging evidence suggesting the presence of a DMN in this species [27]. This indicates that classical psychedelics may hold therapeutic potential for treating anxiety and fear in dogs. Nevertheless, although the evidence in humans is increasingly robust, very few studies have been conducted in animals with the explicit aim of improving their own health. One initiative, the Canine Microdose Project, seeks to assess the safety, efficacy, and potential benefits of microdosing certain naturally occurring psychedelic compounds in dogs [28]. Microdosing refers to the administration of very small quantities of these substances in order to achieve therapeutic effects without perceptual alterations, making it a potentially safer approach when testing in non-human species [28]. However, the legal status of most of these compounds poses significant challenges for regulated research [29]. As an alternative, 1-cyclopropionyl lysergic acid diethylamide (1cp-LSD) [30], a legal analog of LSD, has been proposed as a suitable compound for exploratory research in veterinary behavioral medicine [31,32], although further research is required to clarify mechanisms, safety, and clinical relevance in dogs. Pharmacologically, 1cp-LSD is considered a prodrug of LSD, undergoing in vivo conversion to the parent compound, and exhibits a comparable receptor interaction profile [30]. Although the conversion ratio to LSD in the canine species remains unknown, its action through the same receptors makes 1cp-LSD a safe candidate, without implying any therapeutic superiority over LSD. Additionally, 1cp-LSD is relatively affordable and readily accessible, which facilitates its controlled experimental use.

This study aimed to evaluate the potential welfare-enhancing effects of low doses of 1cp-LSD in shelter dogs within the framework of a randomized blind trial combined with ethological intervention. Our research group has previously reported two single-case studies [31,33] and a prospective cohort of seven dogs and their owners [32], all of which suggested that 1cp-LSD microdosing may contribute to improve canine anxiety. Building on these findings, the present study expands the scope of investigation to the shelter context, providing systematic and controlled data that support the rationale for further exploration of psychedelic-based interventions in veterinary behavioral and welfare science.

## 2. Material and Methods

### 2.1. Subjects and Setting

The study was conducted at the Island Animal Shelter, located in Bañaderos, Gran Canaria (Canary Islands, Spain). The shelter is managed by the Cabildo of Gran Canaria, and explicit authorization was obtained from the institution to carry out the experiment (authorization code AV-FA-61-2024, 26 November). At the time of the study, the facility accommodated 214 dogs housed across 53 individual kennels and 10 communal enclosures, as well as 80 cats kept in two dedicated catteries [34].

From the resident dog population, 20 individuals were selected according to the following inclusion criteria: medium-to-large size, any breed, exhibiting clear signs of behavioral disorders (anxiety, depression, or fear) as assessed by the shelter’s veterinary staff, with no prior ethological interventions and not under judicial custody due to ongoing abuse investigations involving former owners. All dogs included in the study were neutered in accordance with Spanish animal welfare legislation (Law 7/2023, Article 23). No exclusion criteria were applied regarding breed, sex, or length of stay at the shelter.

Of the 20 dogs enrolled, 7 were mixed-breed with phenotype compatible with the Potentially Dangerous Dog (PDD) classification according to Spanish legislation (Real Decreto 287/2002), 5 were mixed-breed of shepherd-type dog (German/Belgian Shepherd-like), and 5 were Canarian Podenco. The mean age was 3.4 ± 2.1 years, and the mean duration of stay at the shelter was 17.6 ± 12.2 months (range 3–48). Fifty percent of the animals were male, and 5 of the 20 were housed in individual kennels. A total of 7 dogs had previously displayed aggressive behavior toward conspecifics, and 4 had attempted to bite shelter staff on at least one occasion. Individual characteristics of the enrolled dogs are presented in Table 1, while their spatial distribution within the facility is provided in S1.

Additionally, 4 extra dogs were pre-selected as reserves in case of adoption during the study period. However, no such event occurred, and the study was completed with the initial cohort of 20 dogs.

### 2.2. Study Design

Group allocation was performed to ensure equivalent baseline welfare and stress levels across the four study arms, with 5 animals per group: (1) control, (2) 1cp-LSD only, (3) ethology-based intervention (EI) only, and (4) combined 1cp-LSD + EI. Pharmacological and ethological treatments were administered over a three-week period. Dogs in the control and 1cp-LSD–only groups continued their usual shelter routines and received no additional training or environmental enrichment. Both groups were monitored through periodic observations only, once a week, with each observation lasting approximately 10 min per animal.

Allocation targeted balance in baseline stress levels across groups (assessed with validated scales described in Section 2.4). When two study dogs cohabited the same enclosure, they were assigned to different arms according to housing logistics. This approach reflected the operational context of the shelter, where the primary objective was to promote animal welfare within the constraints of existing housing conditions. The potential bias from possible cross-exposure between cohabiting dogs is acknowledged and discussed in the Section 5. Among drug-treated dogs, doses were balanced so that mean administered doses did not differ significantly between the 1cp-LSD–only and 1cp-LSD + EI groups.

Two veterinarians with expertise in clinical ethology (blinded) conducted all standardized behavioral/anxiety assessments at baseline (pre-randomization), end of treatment, and end of study. These blinded evaluators also delivered the EI sessions but remained unaware of which EI dogs had received 1cp-LSD. Medication was prepared and administered orally exclusively by two separate (non-blinded) veterinarians.

**Figure 1:**
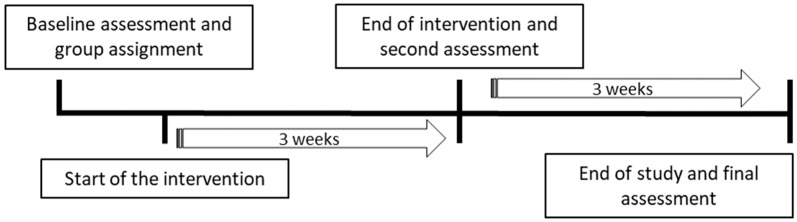
Longitudinal study design with a six-week duration. Dogs (*n* = 20) were allocated to four experimental groups: control, 1cp-LSD treatment, ethology-based intervention (EI), and 1cp-LSD + EI. The intervention phase lasted three consecutive weeks. During this period, 1cp-LSD was administered orally on Mondays, Thursdays, and Sundays, coinciding with the days on which EI sessions were conducted. The start and end of the intervention period are indicated in the timeline. After treatment discontinuation, no pharmacological or ethological interventions were applied until the end of the study, allowing assessment of post-treatment effects.

Drug administration and interventions followed a Monday–Thursday–Sunday schedule over three consecutive weeks, totaling 10 administrations/sessions. This was followed by a three-week period with no direct contact or intervention (periodic observation only) (Figure 1).

All dogs remained in their original housing throughout the study, with no relocation or changes in enclosure assignment before, during, or after the intervention period.

All four field team members completed a validated treatment-expectancy questionnaire at baseline [35], adapted from human clinical research to the veterinary context given the absence of species-specific tools. The instrument comprised 15 questions assessing expectations of treatment influence (Appendix A), scored on a 0–10 Likert scale. Although originally validated in humans, the questionnaire was specifically rephrased to refer to dogs as treatment recipients. To our knowledge, this represents the first application of a treatment-expectancy measure in veterinary behavioral research. Its inclusion was intended to mitigate expectation bias by ensuring balanced attitudes among personnel directly involved in the study. No significant differences were detected between blinded and non-blinded staff, or across intervention modalities (1cp-LSD, EI, combined), in any of the TEX-Q subscales—treatment benefit, positive impact, adverse events, negative impact, process, behavioral control—or in the overall mean score (S2).

### 2.3. Ethical Considerations and Sample Size Rationale

This study was conducted in accordance with the principles of the 3Rs (Replacement, Reduction, and Refinement) and fully complied with Directive 2010/63/EU of the European Parliament on the protection of animals used for scientific purposes, as well as the recommendations of the National Research Council regarding the care and use of animals in research. Approval was obtained from the Animal Experimentation Ethics Committee of the University of Las Palmas de Gran Canaria (Ref. OEBA_ULPGC 02/2024). Permission was also formally granted by the Cabildo of Gran Canaria (Canary Islands, Spain), the authority responsible for the shelter where the study was carried out. Although 1cp-LSD is not an authorized veterinary medicinal product, the protocol was approved on the grounds of its exploratory and welfare-oriented nature, the growing scientific interest in serotonergic psychedelics, and the use of sub-perceptual doses considered non-hallucinogenic [36]. The central ethical justification was that the study did not impose additional suffering but instead sought to explore interventions with potential to improve welfare in dogs living under stressful shelter conditions.

The decision to include five animals per arm was driven by both ethical and methodological constraints. From an ethical perspective, the primary aim was to minimize the number of subjects exposed to experimental conditions while ensuring sufficient representation to generate interpretable exploratory data. Unlike laboratory studies, shelter-based research must balance scientific rigor with the welfare of a limited population of animals living under naturalistic shelter conditions. From a methodological standpoint, a small but balanced cohort across the four arms allowed for randomization and controlled comparisons, while reducing the risk of overburdening shelter resources and staff. A post hoc power analysis, assuming medium to large effect sizes and moderate correlations between repeated measures, indicated that the sample size provided adequate power (≥0.80) to detect group-by-time interaction effects. Moreover, precedent in the literature also indicates that early-stage pharmacological trials in dogs often rely on small cohorts, particularly when assessing novel compounds in exploratory designs [32,37,38]. These considerations justify the limited sample size while underscoring the proof-of-concept character of the present work.

### 2.4. Instruments for Measuring Canine Anxiety

To assess canine anxiety, three validated scales were used: a specific scale to determine the level of separation anxiety (SA) (validated in [39]) and two additional scales to assess the level of general anxiety in shelter dogs: the Multi-Operator Qualitative Behavioral Assessment MO-QBA (validated in [40]) and the Qualitative Behavioral Assessment (QBA) (validated in [13]). The SA scale comprises 17 items that evaluate the frequency and intensity of core behaviors such as vocalization, destructive activity, inappropriate elimination, salivation, and motor activity. Scoring thresholds classify cases from absence to severe separation anxiety, as detailed previously [31,32,33,39].

The MO-QBA comprised a continuous linear scale from 0 (“no stress”) to 5 (“maximum stress”) to capture overall stress intensity, complemented by five subscales (0–5) that assessed sociability, calmness, fear, excitability, and aggressiveness [40]. Items were grouped to create two composite subscales: “Emotional regulation and sociability” (QBA 1), calculated as the sum of sociability and calmness; and “emotional defensiveness and arousal” (QBA 2), calculated as the sum of fear, excitability, and aggressiveness.

The QBA included 20 descriptive items, each rated on a 5-point scale (1 = lowest intensity; 5 = highest intensity). The items captured a broad spectrum of emotional and behavioral expressions, encompassing aggressive, alert, anxious, attention-seeking, bored, comfortable, curious, depressed, excited, explorative, fearful, hesitant, interested, nervous, playful, reactive, relaxed, sociable, stressed, and wary states [13]. Items were grouped to generate for subscales: “Negative emotional reactivity” (QBA 3), “high emotional reactivity” (QBA 4), “positive emotional reactivity” (QBA 5), and “low emotional arousal” (QBA 6) (Appendix B). These items were grouped specifically for the present study to provide a composite measure capturing broader emotional dimensions from the individual behavioral indicators. To ensure consistency across all subscales, QBA1 and QBA5 were reverse scored for subsequent analyses.

All behavioral assessments were conducted by blinded observers, who were unaware of the treatment allocation of the animals. Each evaluation was performed jointly, with both blinded observers completing the scales together for every dog. Assessments were carried out at three time points: prior to the onset of treatment (baseline), immediately upon completion of the intervention period, and three weeks post-treatment (Figure 1). On each evaluation day, all dogs across groups were systematically observed to ensure comprehensive and standardized behavioral assessment. Data were first recorded on paper forms and subsequently digitized by non-blinded investigators.

### 2.5. Interventions

#### 2.5.1. Ethological Intervention

A total of ten dogs were randomly selected and subjected to ethological intervention (EI), five of which received the combined treatment with 1cp-LSD. The interventions were delivered by the two investigators blinded to pharmacological allocation. Over the intervention period, ten ethological sessions were performed. Each session was tailored to the individual dog based on its stress profile, considering species-specific, age-related, and individual behavioral characteristics. Stressors were identified for each dog, and targeted tools and strategies were provided to facilitate stress release and promote the consolidation of adaptive coping mechanisms. Measures to enhance rest were implemented when appropriate, and opportunities for positive intra- and interspecific social interactions were incorporated. This individualized, profile-driven approach ensured that dogs exhibiting anxious, fearful, or depressive behaviors received interventions optimized for their specific welfare needs, fostering the acquisition and maintenance of effective stress management strategies.

The mean duration of each session was 23.5 ± 1.3 min for the EI group and 23.1 ± 1.9 min for the 1cp-LSD+EI group, with no significant difference in time observed between the two groups (*p* = 0.768).

#### 2.5.2. Dosage of 1cp-LSD

1cp-LSD is a lysergamide derivative structurally related to LSD, first made accessible for research purposes in mid-2019 [30]. Following administration, it undergoes hydrolytic cleavage by blood carboxylesterases, resulting in the release of LSD and thereby acting as a prodrug [30]. The active metabolite engages dopaminergic and adrenergic pathways while primarily exerting its effects through agonism at serotonin 5-HT2A receptor [41].

The substance was legally sourced from an online supplier (AlphaChain B.V., Utrecht, The Netherlands), with each pill containing 10 μg of 1cp-LSD L-tartrate. The manufacturer verified the compound’s concentration through quantitative nuclear magnetic resonance (qNMR) spectroscopy.

A total of ten dogs, corresponding to the pharmacological intervention groups (1cp-LSD and 1cp-LSD + EI), received an oral dose of 10 µg of 1cp-LSD per session, administered in solid oral form (tablet), once every three days for a total of ten administrations over the three-week treatment period. To facilitate administration, the substance was concealed within a small portion of wet kibble, which was readily consumed by all dogs without rejection. 1cp-LSD was administered on intervention days by non-blinded investigators, approximately one hour before the onset of ethological sessions. This schedule was designed to ensure that dogs assigned to the combined treatment arm (1cp-LSD + EI) underwent behavioral work while under the pharmacological influence of the compound [42]. On average, the interval between drug administration and the initiation of ethological intervention was 3.04 ± 0.51 h. Dogs in non-pharmacological arms did not receive sham medication; thus, no pharmacological placebo was used.

The selected dose (10 µg) was based on preliminary evidence supporting both safety and preliminary efficacy at this range [32]. Human equivalent dose (HED) was estimated using the Du Bois formula as follows [43]:

BSA (m^2^) = 0.007184 × BW (Kg)^0.425^ × L (cm)^0.725^,

where BSA is defined as body surface area, BW as body weight, and L as length (nose-to-base of the tail). A ratio is then established between the body surface area of a human weighing 80 kg and measuring 180 cm in height, set at 1.99 m^2^, and the BSA of the dog. This ratio was multiplied by the administered dose to obtain the HED. Following this strategy, the administered dose was confirmed to represent a low, sub-perceptual exposure comparable to human microdosing [44]. Importantly, group allocation was explicitly stratified to guarantee the absence of significant differences in dosing between treated animals. Thus, the administered dose was 32.7 ± 3.8 µg for the 1cp-LSD group and 33.9 ± 4.7 µg for the 1cp-LSD + EI group (*p* = 0.662). No adverse effects or short-term side effects were observed in any of the dogs during the treatment period, as assessed through continuous ethological monitoring and routine welfare checks.

#### 2.5.3. Study Timeline

Pharmacological and/or ethological interventions were carried out on 17, 20, 21, 24, 27, and 28 April, and 1, 4, 5, and 8 May 2025, totaling ten sessions distributed across a three-week period. This active phase was subsequently followed by a three-week observation phase with no direct interaction, allowing evaluation of both immediate and sustained effects of the interventions.

### 2.6. Statistical Analysis

Descriptive statistics were computed for all study variables, with means and standard deviations reported for continuous measures. The distribution of the data was examined using the Shapiro–Wilk test to assess normality. Data were analyzed in intragroup comparisons to examine the evolution of dogs over time, and in intergroup comparisons to assess the effects of different interventions. To evaluate intervention effects over time while controlling for interindividual differences, delta (Δ) scores were calculated, representing the change between consecutive time points. This method reduces the impact of baseline variability and facilitates the identification of treatment-related effects. Δ values were subsequently analyzed to assess the significance of changes in canine behavior. Between-group comparisons were conducted using paired *t*-tests or one-way ANOVA when appropriate, followed by Tukey post hoc tests to identify specific group differences. Associations between continuous variables were explored using Pearson correlation coefficients. Statistical significance was set at *p* < 0.05 (two-tailed). Although some results were statistically significant, they should be interpreted cautiously due to limited sample size and the lack of confirmatory replication. All analyses were performed using PASW Statistics (version 19.0, SPSS Inc., Chicago, IL, USA).

## 3. Results

The study was officially launched on 17 April 2025. All twenty enrolled dogs initiated and successfully completed participation within their originally assigned experimental groups, with no instances of attrition, crossover, or protocol violation. Full compliance with the scheduled intervention plan was achieved, and no adverse events or unforeseen contingencies were recorded throughout the study period. The trial was therefore conducted in strict accordance with the predefined protocol and reached its planned completion on 2 June 2025.

### 3.1. Environmental Influence on Baseline Animal Behavior

Environmental factors related to the shelter habitat were examined to assess their potential influence on behavioral outcomes at baseline. The next findings reflect baseline behavioral variation in relation to environmental and social factors without implying causal effects. Table 2 summarizes the bivariate correlations between contextual variables (age, time in shelter, enclosure size, and available space per animal) and the behavioral indicators derived from the questionnaires. Among these variables, time in shelter showed significant associations with several endpoints. Specifically, longer shelter residence was negatively correlated with stress (Pearson r = −0.572, *p* = 0.008), fear (Pearson r = −0.665, *p* = 0.001), QBA 2 (emotional defensiveness and arousal; Pearson r = −0.463, *p* = 0.040), and QBA 3 (negative emotional reactivity; Pearson r = −0.555, *p* = 0.011). Conversely, it was positively correlated with sociability (Pearson r = 0.550, *p* = 0.012), QBA 1 (emotional regulation and sociability; Pearson r = 0.485, *p* = 0.030), and QBA 5 (positive emotional reactivity; Pearson r = 0.547, *p* = 0.013). These findings suggest that the duration of stay in the shelter may play a modulatory role in the emotional and social expression of dogs, potentially reflecting adaptive processes to the kennel environment. It should be noted that no significant differences were observed in the mean length of stay in the shelter across groups (*p* = 0.843).

Associations between baseline behavioral indicators and key dichotomous contextual variables—including housing type, cohousing with other study dogs, being taken out of the enclosure, and the presence of behavioral issues with conspecifics—are summarized in S3. Sociability scores were associated with several contextual variables. Dogs that were taken out of the enclosure exhibited higher sociability compared to those that were not taken out (2.7 ± 0.3 vs. 0.6 ± 0.2; *p* = 0.001). Lower sociability was observed in dogs cohoused with other study dogs compared to those that were not (1.3 ± 0.3 vs. 3.5 ± 0.6; *p* = 0.002), and in dogs with behavioral issues in interactions with other dogs compared to those without such issues (1.6 ± 0.3 vs. 3.1 ± 0.5; *p* = 0.011). Dogs in group housing displayed higher fear compared to those in individual housing (3.3 ± 0.4 vs. 0.8 ± 0.3; *p* = 0.004). Higher fear was also observed in dogs cohoused with other study dogs compared to those that were not (4.0 ± 0.6 vs. 1.5 ± 0.6; *p* = 0.004). Dogs that were taken out of the enclosure exhibited lower fear compared to those that were not (2.0 ± 0.4 vs. 4.8 ± 0.2; *p* < 0.001), and dogs with behavioral issues in interactions with other dogs also showed lower fear than those without such issues (3.5 ± 0.4 vs. 1.5 ± 0.7; *p* = 0.013). Additional associations between contextual variables and baseline behavioral indicators are presented in S3. No significant differences were observed with respect to sex or attempted bites toward staff. Overall, baseline behavioral variation in dogs was closely associated with environmental and social factors, reflecting the influence of housing, handling, and conspecific interactions.

### 3.2. Intra-Group Behavioral Analysis

Intra-group analyses are presented in Table 3, which summarizes mean scores and ranges (in parentheses) for behavioral and emotional measures before treatment, after treatment, and at the end of the study. No significant changes were observed in any behavioral variables within the control group. In the 1cp-LSD-treated group, stress levels decreased significantly from baseline to the end of treatment (3.5 vs. 3.0; *p* = 0.047). No significant differences were found in any behavioral measures in the ethological intervention (EI) group. In the combined 1cp-LSD + EI group, aggression decreased significantly during the initial treatment period (baseline vs. end of treatment; 2.8 vs. 1.2; *p* = 0.030), while both calmness and QBA 1 (emotional regulation and sociability) increased significantly from the end of treatment to the end of the study (1.6 vs. 2.7, *p* = 0.004; and 3.6 vs. 5.0, *p* = 0.005, respectively). Although these observations cannot establish causality, the data suggest that 1cp-LSD administration, alone or in combination with EI, may be associated with reductions in stress and aggression, and increases in calmness and emotional regulation, indicating a potential modulatory effect on behavioral and emotional profiles in shelter dogs. These findings are observational and do not allow for causal inferences, but they provide valuable evidence on how different types of interventions may be associated with behavioral and emotional changes in shelter dogs.

### 3.3. Inter-Group Behavioral Analyses

Inter-group behavioral analyses were conducted to compare the trajectories of the different treatment conditions over time. These comparisons are essential to determine whether the observed changes within each group represent specific intervention-related effects or reflect broader patterns unrelated to treatment allocation.

**Figure 2:**
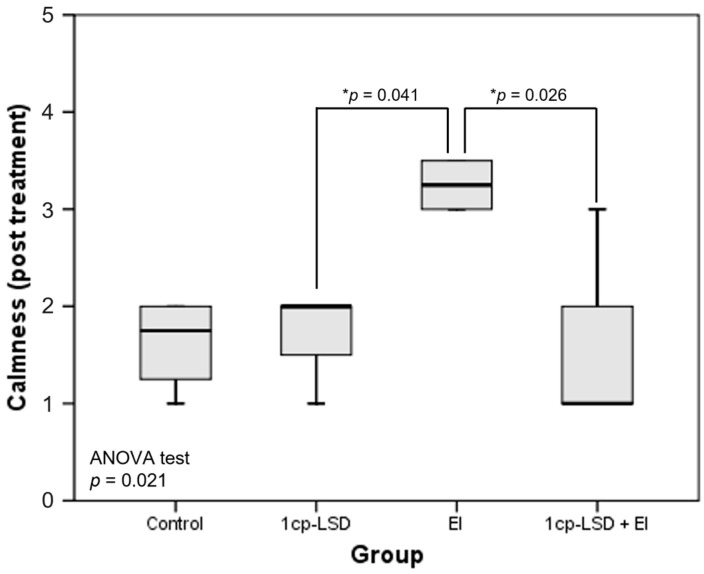
Boxplot showing significant variations in the calmness at the end of treatment, across the different experimental groups: Control, 1cp-LSD, ethology-based intervention (EI), and 1cp-LSD + EI. The horizontal line within each box indicates the median; the lower and upper edges represent the 25th and 75th percentiles, respectively; and the whiskers denote the full data range. * Tukey post hoc test.

Calmness levels differed significantly at the end of the treatment period, being higher in the group of dogs that received EI (Figure 2; *p* = 0.021). Specifically, pairwise comparisons revealed differences between the 1cp-LSD and EI groups (1.7 vs. 3.0; *p* = 0.041), as well as between the 1cp-LSD + EI and EI groups (1.6 vs. 3.0; *p* = 0.026). These differences were not observed at the end of the study. No other significant inter-group differences were detected for the remaining behavioral endpoints, either at the end of treatment or at the conclusion of the study.

**Figure 3:**
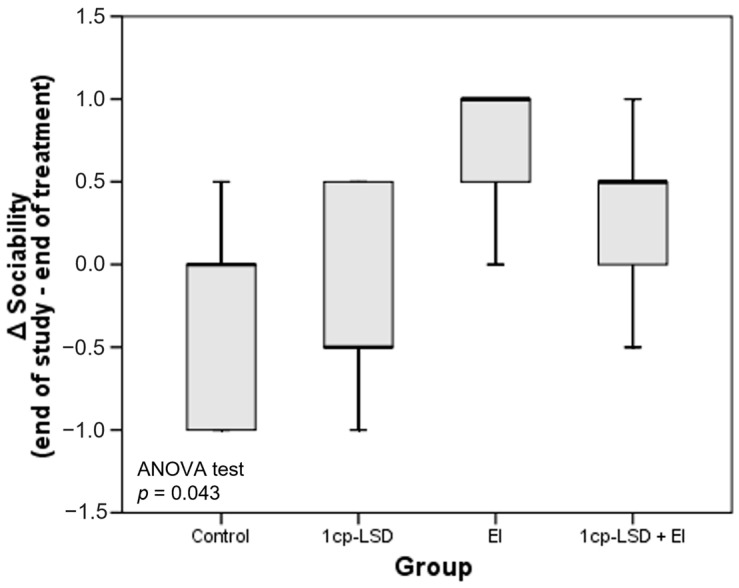
Boxplot illustrating changes in sociability scores across the experimental groups: Control, 1cp-LSD, ethology-based intervention (EI), and 1cp-LSD + EI. Delta score (Δ) represent the change in sociability after treatment cessation, calculated as the difference between the end of the study and the end of the treatment period. Positive delta values indicate a sustained improvement in sociability over time, values close to zero indicate no change, and negative values indicate a reduction in sociability following treatment withdrawal. The horizontal line within each box indicates the median; the lower and upper edges represent the 25th and 75th percentiles, respectively; and the whiskers denote the full data range.

Additional analyses were performed using delta scores, calculated as the change between the start of intervention and end of treatment, and between the end of treatment and the end of the study. This approach provides insight into the persistence or evolution of behavioral and emotional responses once the interventions were completed, highlighting whether observed effects were transient or sustained over time. Significant differences in sociability levels were observed in the period between the end of treatment and the end of the study, with apparently higher values in the group of dogs that received EI (Figure 3; *p* = 0.043). However, post hoc comparisons did not reveal specific significant differences in pairwise group analyses.

**Figure 4:**
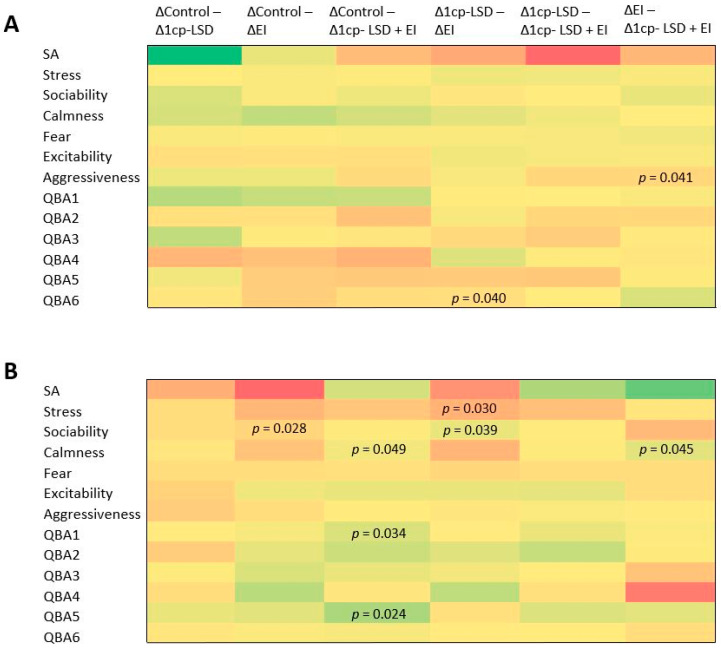
Heatmap representation of changes in the composite anxiety-related across treatment groups. Colors represent the direction and magnitude of change, with red shades indicating improvement and green shades indicating worsening. Numeric values were omitted for clarity; only statistically significant *p* values (paired *t*-test, *p* < 0.05) are displayed within the cells. Panel (**A**) shows the mean change (Δ) from baseline to the end of treatment for each group. Panel (**B**) shows the mean change between the end of the study and the end of treatment, illustrating post-treatment trends after intervention cessation. To ensure that higher values consistently reflect greater anxiety or emotional disturbance, positively valanced items (e.g., relaxed, playful, sociable, comfortable, curious, explorative) were reverse coded prior to summation. This transformation allowed the score to be interpreted in a unified direction, improving its interpretability in group comparisons.

Following this strategy, pairwise analyses were then performed across the four treatment conditions (six comparisons in total) to identify potential differences in the magnitude of behavioral change (Figure 4). Aggressiveness significantly increased during the period between the beginning of the study and the end of treatment, specifically between the EI and 1cp-LSD + EI groups, with a delta of 1.60 points (Figure 4A; *p* = 0.041), indicating that aggressiveness was significantly higher in the group of dogs that received the EI treatment. Similarly, QBA 6 (low emotional arousal) significantly increased over the same time period, specifically between the 1cp-LSD and EI groups, with a delta of 1.20 points (*p* = 0.040), indicating that this variable was significantly higher in the group of dogs that received 1cp-LSD. These findings suggest that treatments applied in isolation may have less adaptive or even counterproductive effects compared to combined strategies, highlighting the potential value of integrated interventions.

Finally, during the period between the end of treatment and the end of the study (Figure 4B), sociability levels were lower in the control group compared to the EI group (delta = −1.20; *p* = 0.028), as well as in the 1cp-LSD group compared to the EI group (delta = −1.10; *p* = 0.039). Calmness levels were lower in the control and EI groups compared to the group of animals that received the combined treatment (*p* = 0.049 and 0.045, respectively). The control group also exhibited lower QBA 1 (emotional regulation and sociability) and QBA 5 (positive emotional reactivity) scores compared to the animals in the 1cp-LSD + EI group (*p* = 0.034 and 0.024, respectively). Overall, these findings indicate that the combined 1cp-LSD and ethological intervention produced the most pronounced improvements in sociability, calmness, and positive emotional reactivity, compared to either intervention alone or no intervention.

## 4. Discussion

This study examined behavioral and emotional responses in shelter dogs across different interventions. While individual treatments produced limited changes, the combined 1cp-LSD and ethological intervention consistently enhanced sociability, calmness, and positive emotional reactivity, suggesting that integrated approaches may be particularly effective in supporting adaptive behavioral profiles in shelter environments.

Before considering treatment effects, it is essential to contextualize these findings within the baseline behavioral profiles of the dogs. The demographic profile of our cohort reflects that reported in other shelters: adult, large-sized, mixed-breed animals [1]. However, it is noteworthy that there was a high proportion of Canarian Podenco dogs, primarily used for hunting. Importantly, the current Animal Welfare Law in Spain (Law 7/2023, of March 28) excludes hunting dogs, despite estimates that they represent approximately 13% of the dog population in Spanish shelters [3], a discrepancy difficult to justify given the observed breed composition. Analyses of the initial observations indicated that environmental factors exerted a measurable influence on sociability, fear, and emotional reactivity in dogs prior to any intervention. In general, length of stay in the shelter has been associated with the development of certain behavioral challenges, including increased arousal and difficulties in relaxation [9]. In the present series, dogs appeared to adapt progressively over time, showing reduced stress and fear levels and increased sociability the longer they remained in the shelter. Previous studies have identified several factors potentially affecting shelter dog welfare, including inadequate indoor housing during extreme temperatures, insufficient bedding, nutritionally suboptimal diets, limited socialization programs, the absence of structured adoption strategies, and staff lacking formal training in animal behavior and welfare [45], as well as the available space per animal, limited access to toys, or intermittent, excessive barking noise [10]. Although the average space per animal was small in the present series (median = 6.2 m^2^), 15 of the 20 dogs (75%) were regularly allowed out of their enclosures prior to the start of the study, suggesting that the animals are likely receiving attentive care and an environment that supports their behavioral and emotional well-being. This observation is noteworthy, as it reflects the efforts of shelter staff despite the challenging conditions and workload. Baseline observations highlighted substantial individual and contextual variability in dog behavior. Factors such as housing type, interactions with conspecifics, and environmental handling were associated with differences in sociability and fear. Importantly, these findings are observational and do not imply causality. From an ethological perspective, the results likely reflect individual predispositions, social hierarchies, and stress-related dynamics inherent to the shelter environment.

Given the observed baseline variability, structured interventions (ethological and/or pharmacological) represent a promising approach to reducing stress and enhancing sociability and emotional regulation in shelter dogs. Ethological interventions for shelter dogs encompass human interaction (e.g., outings, fostering), sensory enrichment (e.g., music, pheromones), behavioral modification, and animal-assisted activities [19,46]. These approaches have been associated with reduced stress and improved welfare—through decreases in cortisol, arousal, and vocalization, and increases in relaxation [47]—as well as with reductions in undesirable behaviors such as aggression or reactivity [48]. In our study, ethological intervention produced significant improvements, with dogs showing increased calmness and sociability over time. The presence of inter-group differences and significant delta comparisons suggests that EI-related effects may be better characterized as relative improvements with respect to other conditions (or in temporal trajectories) rather than large absolute changes within individuals over the short study interval. Accordingly, these findings should be interpreted cautiously and underscore the value of larger samples, longer follow-up, and longitudinal mixed-effects modeling to fully characterize EI effects. However, many benefits, particularly those derived from brief or situational interventions, tended to fade once dogs returned to the shelter environment, underscoring the need for strategies with more sustained effects [46,47]. Sustaining improvements in behavior is particularly relevant, as evidence indicates that dogs benefiting from such interventions are more likely to be adopted and successfully rehomed [46].

The therapeutic potential of psychedelic substances in the field of psychiatry has been known for decades. Unlike conventional pharmacological treatments, which are frequently associated with high rates of relapse, dependence, tolerance, and habituation to drugs with limited safety margins [49,50,51], psychedelics appear to offer certain therapeutic advantages. In addition to being regarded as safe when administered in therapeutic contexts [52,53], psychedelics are non-addictive [54] and sustain their therapeutic effects even after treatment discontinuation [55]. Although systematic data in dogs are currently lacking, preclinical evidence from animal models suggests that LSD exhibits at most weak reinforcing effects and does not produce clear addiction-like patterns, indicating a low dependence potential for classic serotonergic psychedelics [56]. However, data on chronic exposure remain limited. An additional advantage of this compound is its practical feasibility in shelter settings, as the monthly treatment protocol used in this study (10 administrations of 10 µg each, administered every three days) represents an estimated cost of around 15 € per animal, and the substance is readily accessible in countries where its use is legal. These characteristics make them particularly suitable in the context of veterinary pharmacology, where ethical considerations, animal welfare and economically sustainable interventions are paramount. In the present study, we observed a reduction in stress levels in the group of animals treated with 1cp-LSD. Following treatment discontinuation, no statistically significant changes were detected, suggesting that some beneficial effects may persist for a limited period, although certain behaviors showed non-significant trends toward baseline levels. Compared to the control group, no significant differences were observed in the behavioral endpoints assessed. It should be noted that the therapeutic effects of psychedelics in human medicine are maximized when treatment is carried out in therapeutic settings [53] and combined with psychological support [55]. Moreover, patients typically undergo psychedelic treatments with a clear therapeutic intention and a motivating goal [53,55]. In contrast, in veterinary medicine, animals are exposed to treatment inadvertently, which makes the use of non-hallucinogenic doses indispensable to safeguard animal welfare. Taken together, the absence of psychological support and the lack of intentionality in treatment—both for obvious reasons in the veterinary context—may help explain the results observed in the present study. Nevertheless, the sustained anxiolytic effect of psychedelics has been previously documented in dogs [31,32]. Given the experimental design and the limited number of animals per group, the current results cannot demonstrate causality and should be interpreted with caution.

In a previous study, the relationship established between the owner and the animal was shown to condition the success of such treatments [32], an influence deliberately excluded in the present study. Nonetheless, prior evidence indicates that animal welfare and human well-being are closely interrelated [57], which helps to explain the beneficial effects observed with structured ethological interventions [46]. Although behavioral interventions performed by ethologists are not cognitively equivalent to psychological treatments, they represent an essential component for improving animal welfare, particularly in shelter settings [19,46], as observed in the present study with respect to calmness and sociability. Individually, both ethological interventions and 1cp-LSD treatment appear to contribute to enhancing dog welfare [19,31,32,46]. When administered together, the current results indicate that the combined treatment improves behavioral outcomes compared to the control group and to the group receiving only ethological intervention, although no significant differences were observed when comparing the two active treatments. Importantly, the therapeutic effect persisted for one month after the interventions concluded.

Overall, these findings underscore the complex interplay between individual predispositions, environmental factors, and structured interventions in shaping shelter dog behavior. While baseline variability highlights the influence of housing, social dynamics, and handling, the present study demonstrates that ethological and pharmacological approaches—particularly when combined—can promote sustained improvements in key behavioral and emotional domains. This emphasizes the potential value of integrated strategies for enhancing welfare in shelter environments, while also highlighting the need for further research with larger cohorts and extended follow-up to fully understand and optimize these effects.

## 5. Strengths and Limitations

This study presents several limitations that should be considered when interpreting the results. First, there are methodological constraints related to inter-observer bias, as assessments were conducted jointly. In addition, the life history of the dogs prior to their admission to the shelter (e.g., experiences of abuse, age at abandonment, or level of socialization) was unknown, representing a major source of uncontrolled variability. The type of stress displayed by the animals was also heterogeneous, and pre-study routines, which could have had either positive or negative effects, were not systematically recorded. Potential cross-exposure effects between cohabiting dogs must also be acknowledged, as behavioral changes in treated animals could have indirectly influenced kennel-mates assigned to other groups. However, further stratified or post hoc re-analyses to isolate this effect were not feasible, as they would have substantially reduced group sizes and compromised baseline comparability, which was a core criterion of the original allocation strategy. Potential contamination by parallel management practices must also be acknowledged, since routine shelter activities (e.g., walks, handling) may have attenuated differences between groups. Second, the experimental design presents statistical limitations. The small sample size (*n* = 20, divided into four groups) restricted statistical power, particularly for within-group analyses. High interindividual variability implies that, even when some dogs showed marked improvements, group averages may have masked such changes. Moreover, floor and ceiling effects limited the scope for improvement in subjects who started with already extreme scores. Measurement timing constituted another constraint, as some transient effects may have emerged only at intermediate points and thus were not captured by within-group comparisons. The ethological interventions may also have been limited in duration or intensity, reducing the likelihood of producing sustained individual-level changes, even though relative differences between groups could still be observed. Finally, pharmacological limitations should be taken into account. No specific data are currently available on the dose–time curve of 1cp-LSD in dogs, and dosage estimations were extrapolated from human references [42]. This absence of species-specific information constrains the accuracy of pharmacodynamic interpretations. Although these approaches allow only approximate estimations, they fail to capture species-specific differences in absorption, metabolism, and receptor sensitivity. This highlights the importance of conducting pharmacokinetic studies to establish appropriate dosing protocols in dogs. Moreover, the lack of longitudinal data means that potential long-term or cumulative effects of repeated low-dose exposure to 1cp-LSD in dogs cannot yet be fully excluded and warrant careful investigation. Breed-related factors may also modulate both the pharmacokinetics and pharmacodynamics of 1cp-LSD, which could partly explain the variability in treatment responses observed in this study. Future research should focus on establishing the pharmacokinetic and pharmacodynamic profiles of 1cp-LSD in dogs, with particular attention to breed-related variability, in order to define safe and effective dosing strategies. Expanding sample sizes, extending follow-up periods, and applying longitudinal analytical approaches will help to capture both transient and sustained effects. Importantly, studies conducted in shelter environments should continue to integrate pharmacological and ethological interventions under controlled conditions, as this translational framework is essential for improving the welfare of dogs living in kennels and for advancing evidence-based practices in veterinary behavioral medicine.

In contrast to the aforementioned limitations, this study also presents several strengths that reinforce its scientific relevance. To our knowledge, it is the first investigation exploring the potential therapeutic effects of 1cp-LSD on canine anxiety in shelter environments. The longitudinal design allowed for the assessment of both short- and medium-term outcomes, offering insight into the persistence of behavioral changes beyond the active treatment phase. Although the sample size was limited due to ethical and design-related constraints, the data generated provide valuable preliminary evidence and are comparable in scale to experimental studies of novel pharmacological agents in companion animals [37,58,59]. All groups shared comparable baseline behavioral conditions, with no significant differences detected between them. Similarly, no significant variations were observed in either dosing or intervention duration across groups, thereby ensuring robust inter-group comparability. Additional methodological strengths further enhance the robustness of the findings. The ethological interventions were carried out by veterinary professionals specialized in animal behavior. These interventions were conducted under blinded conditions, with the two independent evaluators required to reach consensus in their assessments, thereby reducing observer bias. The controlled environment of the shelter minimized external confounders, while the stability of the groups across the trial ensured consistency throughout the experimental period. Finally, the use of validated anxiety assessment scales guaranteed objective and standardized measurements of behavioral change, and the inclusion of a treatment expectation scale provided further depth by capturing investigator-related influences.

## 6. Conclusions

The combined administration of 10 µg of 1cp-LSD and ethological intervention over a three-week period significantly improved specific items related to the welfare of shelter dogs, outperforming both pharmacological treatment and ethological intervention when applied separately. Moreover, these improvements persisted for an additional three weeks after treatment cessation. These findings open a new line of research for managing stress and anxiety in shelter dogs, with the potential to enhance animal welfare and increase adoption rates. Due to the limitations of this research, future studies should involve larger cohorts and focus on specific behavioral pathologies, thereby advancing evidence-based interventions in veterinary behavioral medicine.

## Figures and Tables

**Table 1:** Descriptive data of the canine subjects included in the study.

ID	Breed *	Age (Months)	Weight (kg)	Body Length (cm)	Sex	Time at Shelter (Months) **	Housing Type	Area Per Animal (m^2^)	Behavioral Issues with Other Dogs ***	Attempted Bite Toward Staff
001 ^(1)^	C-Pod	85	26.8	83.0	Male	48	Group	6.2	No	No
002 ^(1)^	Mx-PDD	52	25.7	73.0	Female	34	Individual	12.5	Yes	No
003 ^(2)^	Mx-PDD	59	25.3	83.0	Female	29	Group	6.2	No	No
004 ^(3)^	Mx-PDD	79	32.3	85.0	Female	29	Individual	2.3	Yes	No
005 ^(4)^	Mx-PDD	87	25.0	73.0	Female	28	Individual	2.3	No	Yes
006 ^(3)^	Mx-Shep	19	17.0	78.5	Male	6	Group	3.1	No	No
007 ^(1)^	C-Pod	70	19.4	78.0	Male	10	Group	33.6	Yes	No
008 ^(4)^	Mx	25	16.4	66.0	Female	8	Group	3.1	No	No
009 ^(2)^	Mx-PDD	50	21.5	70.0	Male	28	Group	6.2	No	Yes
010 ^(4)^	Mx-PDD	38	25.5	75.0	Female	28	Group	6.2	No	Yes
011 ^(2)^	Mx-Terr	39	17.8	71.0	Male	18	Individual	2.3	Yes	No
012 ^(3)^, ^(a)^	Mx-Bard	30	30.0	89.0	Male	16	Group	56.0	Yes	No
013 ^(4)^, ^(b)^	Mx-PDD	40	17.8	71.0	Female	16	Individual	2.3	Yes	Yes
014 ^(3)^	C-Pod	36	17.4	82.0	Female	7	Group	33.6	No	No
015 ^(2)^	Mx-Shep	13	12.0	82.0	Female	10	Group	20.4	No	No
016 ^(4)^	Mx-Shep	13	11.5	87.0	Male	10	Group	9.0	No	No
017 ^(3)^	Mx-Shep	13	15.0	89.0	Male	10	Group	20.4	Yes	No
018 ^(1)^	Mx-Shep	13	12.8	83.0	Male	10	Group	20.4	No	No
019 ^(1)^	C-Pod	85	16.4	87.0	Female	3	Group	6.2	No	No
020 ^(2)^	C-Pod	38	20.8	87.0	Male	3	Group	6.2	No	No

* Standardized abbreviations were used. Mixed-breed dogs (Mx) were categorized according to their predominant phenotypic traits as follows: C-Pod, Canarian Podenco; Mx-PDD, mixed-breed with phenotype compatible with the Potentially Dangerous Dog (PDD) classification according to Spanish legislation (Real Decreto 287/2002); Mx-Shep, mixed-breed of shepherd-type dog (German/Belgian Shepherd-like); Mx-Bard, mixed-breed of Bardino Majorero; Mx-Terr, mixed-breed of terrier-type dog; Mx, mixed-breed with no predominant breed phenotype. ** Duration of the dog’s stay in the shelter until the date of data collection. *** Presence of inter-dog aggression or other behavioral issues toward conspecifics. ^(1)^ Dog belonging to the 1cp-LSD–treated group; ^(2)^ dog belonging to the 1cp-LSD + ethological intervention group; ^(3)^ dog belonging to the ethological intervention group; and ^(4)^ dog belonging to the control group. ^(a)^ Dog adopted and later returned to the shelter. ^(b)^ Dog diagnosed with cranial cruciate ligament rupture.

**Table 2:** Bivariate correlations between contextual variables and behavioral indicators at baseline (*n* = 20). Significant results (*p* < 0.05) are highlighted in bold.

	Age	Time in Shelter	Enclosure Size	Space Per Animal
Separation anxiety ^a^				
Pearson r	0.027	0.093	−0.250	−0.101
*p* value	0.929	0.763	0.410	0.742
Stress ^b^				
Pearson r	−0.385	−0.572	0.251	0.182
*p* value	0.093	**0.008**	0.285	0.443
Sociability ^b^				
Pearson r	0.038	0.550	−0.083	0.112
*p* value	0.873	**0.012**	0.727	0.638
Calmness ^b^				
Pearson r	0.111	0.254	0.108	0.065
*p* value	0.642	0.280	0.650	0.786
Fear ^b^				
Pearson r	−0.268	−0.665	0.227	0.046
*p* value	0.252	**0.001**	0.337	0.847
Excitability ^b^				
Pearson r	0.244	0.061	0.018	0.146
*p* value	0.300	0.799	0.939	0.539
Aggressiveness ^b^				
Pearson r	0.050	−0.203	−0.355	−0.357
*p* value	0.835	0.391	0.125	0.122
QBA 1				
Pearson r	0.092	0.485	0.018	0.107
*p* value	0.700	**0.030**	0.941	0.654
QBA 2				
Pearson r	−0.053	−0.463	−0.023	−0.087
*p* value	0.826	**0.040**	0.923	0.714
QBA 3 ^c^				
Pearson r	−0.207	−0.555	0.052	−0.130
*p* value	0.380	**0.011**	0.828	0.584
QBA 4 ^c^				
Pearson r	−0.110	0.116	−0.064	0.048
*p* value	0.644	0.626	0.790	0.842
QBA 5 ^c^				
Pearson r	0.167	0.547	0.129	0.265
*p* value	0.481	**0.013**	0.586	0.259
QBA 6 ^c^				
Pearson r	0.165	0.123	−0.181	−0.167
*p* value	0.488	0.605	0.446	0.481

Abbreviations: QBA, quality behavior assessment. QBA 1 (emotional regulation and sociability): sum of sociability and calmness; QBA 2 (emotional defensiveness and arousal): sum of fear, excitability, and aggressiveness; QBA 3 (negative emotional reactivity): sum of items 1, 11, 12 and 20 (aggressive, fearful, hesitant, and cautious); QBA 4 (High emotional reactivity): sum of items 2, 3, 4, 9, 14, 16, and 19 (alert, anxious, attention-seeking, excited, nervous, reactive, and stressed); QBA 5 (positive emotional reactivity): sum of items 6, 7, 10, 13, 15, 17 and 18 (comfortable, curious, exploratory, interested, playful, relaxed, and sociable); and QBA 6 (low emotional arousal): sum of items 5 and 8 (bored and depressed). ^a^ *Journal of Veterinary Behavior* (2006) 1, 109–120. ^b^ *Applied Animal Behaviour Science* 213 (2019) 107–116. ^c^ *PLoS ONE 14:10* (2019) e0212652.

**Table 3:** Mean scores and ranges (in parentheses) for behavioral and emotional measures in shelter dogs before treatment, after treatment, and at the end of the study. Significant results (*p* < 0.05) are highlighted in bold.

Group	Pre Treatment	Post Treatment	End of Study	*p* Value *	*p* Value **
Control					
SA ^a^	12 (3.5–24.5)	6.9 (3.0–11.5)	8.9 (3.5–14.0)	0.401	0.482
Stress ^b^	3.1 (2.0–4.0)	2.9 (2.0–4.3)	3.2 (2.0–4.5)	0.338	0.446
Sociability ^b^	2.6 (2.0–3.0)	2.4 (1.0–4.0)	2.1 (1.0–3.0)	0.621	0.374
Calmness ^b^	2.2 (0–5.0)	1.9 (1.0–3.0)	2.1 (1.0–3.5)	0.716	0.587
Fear ^b^	2.0 (0–4.0)	1.6 (0–4.0)	1.7 (0–4.0)	0.477	0.815
Excitability ^b^	2.7 (1.0–4.0)	3.6 (2.0–5.0)	3.0 (1.0–4.5)	0.221	0.573
Aggressiveness ^b^	1.4 (0–4.0)	1.2 (0–2.5)	1.4 (0–4.0)	0.717	0.587
QBA 1	4.8 (2.0–8.0)	4.3 (2.0–6.0)	4.2 (2.5–5.5)	0.582	0.861
QBA 2	6.1 (2.0–10)	6.4 (3.0–9.0)	6.1 (4.5–9.0)	0.843	0.786
QBA 3 ^c^	11.2 (6.0–14)	10. 0 (7.5–12)	8.6 (4.5–11.5)	0.470	0.357
QBA 4 ^c^	20.7 (12–26)	21.3 (15–23.5)	21.5 (19.5–24)	0.690	0.893
QBA 5 ^c^	18.2 (16–21)	20.4 (15–26.5)	19.1 (14–25.5)	0.194	0.173
QBA 6 ^c^	4.4 (2.0–9.0)	5.4 (2.0–10)	5.2 (2.0–9.5)	0.266	0.859
1cp-LSD					
SA	7.0 (0.8–18)	6.3 (3.5–10)	7.5 (1.5–15.5)	0.872	0.655
Stress	3.5 (3.0–4.0)	3.0 (2.3–4.0)	3.4 (3.0–4.0)	**0.047**	0.080
Sociability	1.8 (0–4.0)	2.3 (1.0–3.0)	2.1 (1.5–2.5)	0.413	0.541
Calmness	1.2 (0–3.0)	1.7 (1.0–2.0)	2.2 (0–4.0)	0.473	0.519
Fear	3.0 (0–5.0)	2.5 (1.0–4.0)	2.7 (1.5–3.5)	0.519	0.688
Excitability	4.2 (3.0–5.0)	3.9 (3.0–5.0)	3.2 (2.0–5.0)	0.529	0.080
Aggressiveness	0.8 (0–3.0)	0.8 (0–3.0)	0.8 (0–3.5)	0.996	0.996
QBA 1	3.0 (0–4.0)	4.0 (3.0–5.0)	4.3 (1.5–6.0)	0.129	0.675
QBA 2	8.0 (5.0–13)	7.2 (5.0–10)	6.7 (4.0–11.5)	0.685	0.561
QBA 3	11.8 (6.0–15)	119 (9.0–15)	11.3 (7.0–14)	0.943	0.618
QBA 4	24.2 (20–33)	21.4 (19–25)	21.7 (14–28)	0.122	0.903
QBA 5	17.6 (13–20)	19.9 (15–24)	19.7 (16.5–24)	0.079	0.885
QBA 6	2.4 (2.0–3.0)	2.6 (2.0–3.0)	2.7 (2.0–5.0)	0.374	0.866
Ethology-based intervention (EI)					
SA	7.2 (3.0–14)	2.9 (1.5–4.5)	2.6 (1.5–3.5)	0.310	0.783
Stress	3.4 (1.0–5.0)	3.1 (1.0–4.0)	2.7 (0.5–4.0)	0.675	0.160
Sociability	2.4 (1.0–5.0)	2.1 (1.0–4.0)	3.0 (2.0–5.0)	0.634	0.053
Calmness	2.0 (1.0–4.0)	3.0 (2.0–3.5)	2.8 (2.0–4.0)	0.129	0.717
Fear	2.8 (0–5.0)	2.2 (0–4.0)	2.4 (0.5–4.0)	0.070	0.374
Excitability	2.8 (2.0–4.0)	2.6 (2.0–3.0)	3.0 (2.0–4.0)	0.749	0.528
Aggressiveness	1.0 (0–3.0)	1.0 (0–2.0)	1.3 (0–3.5)	0.991	0.501
QBA 1	4.4 (2.0–8.0)	5.1 (4.0–7.5)	5.8 (4.5–8.0)	0.454	0.226
QBA 2	6.6 (4.0–11)	5.8 (3.0–8.0)	6.7 (5.0–11)	0.374	0.295
QBA 3	11.6 (5.0–17)	10.2 (4.0–17)	10.3 (4.5–15.5)	0.245	0.880
QBA 4	22.6 (13–25)	18.4 (13–26)	20.8 (13.5–28)	0.295	0.453
QBA 5	20.2 (15–27)	20.3 (17–28)	20.3 (17–23.5)	0.970	0.906
QBA 6	4.2 (2.0–7.0)	3.2 (2.0–7.0)	3.7 (2.0–8.5)	0.089	0.142
1cp-LSD + EI					
SA	16. 2 (9.0–24)	8.0 (4.0–12.5)	11.7 (10.5–14)	0.302	0.132
Stress	3.5 (0.5–5.0)	3.1 (1.5–4.5)	3.0 (1.0–4.0)	0.338	0.621
Sociability	2.0 (0–4.0)	2.0 (0–4.0)	2.3 (1.0–4.0)	0.990	0.305
Calmness	1.0 (0–2.0)	1.6 (1.0–3.0)	2.7 (1.5–4.5)	0.305	**0.004**
Fear	3.0 (1.0–5.0)	2.5 (0–4.5)	2.8 (1.0–4.0)	0.374	0.374
Excitability	3.6 (3.0–5.0)	3.3 (1.0–5.0)	3.8 (2.5–5.0)	0.704	0.460
Aggressiveness	2.8 (1.0–4.0)	1.2 (0.5–2.5)	1.9 (1.0–3.5)	**0.030**	0.135
QBA 1	3.0 (0–6.0)	3.6 (1.0–6.0)	5.0 (2.5–7.5)	0.235	**0.005**
QBA 2	9.4 (5.0–14)	7.0 (3.5–9.5)	8.5 (4.5–10)	0.166	0.184
QBA 3	12.8 (6.0–17)	10.8 (5.0–13)	10.5 (6.0–14.5)	0.099	0.810
QBA 4	24.6 (20–32)	21.6 (17–24)	22.1 (18–25)	0.165	0.740
QBA 5	18.0 (10–24)	17.9 (10–25)	19.2 (12.5–25)	0.939	0.062
QBA 6	2.6 (2.0–4.0)	2.3 (2.0–3.5)	2.9 (2.0–4.0)	0.634	0.324

Abbreviations: SA, separation anxiety; QBA, quality behavior assessment. QBA 1 (emotional regulation and sociability): sum of sociability and calmness; QBA 2 (emotional defensiveness and arousal): sum of fear, excitability, and aggressiveness; QBA 3 (negative emotional reactivity): sum of items 1, 11, 12 and 20 (aggressive, fearful, hesitant, and cautious); QBA 4 (High emotional reactivity): sum of items 2, 3, 4, 9, 14, 16, and 19 (alert, anxious, attention-seeking, excited, nervous, reactive, and stressed); QBA 5 (positive emotional reactivity): sum of items 6, 7, 10, 13, 15, 17 and 18 (comfortable, curious, exploratory, interested, playful, relaxed, and sociable); and QBA 6 (low emotional arousal): sum of items 5 and 8 (bored and depressed). * Student *T*-test (pre vs. post treatment). ** Student *t*-test (post treatment vs. end of study). ^a^ *Journal of Veterinary Behavior* (2006) 1, 109–120. ^b^ *Applied Animal Behaviour Science* 213 (2019) 107–116. ^c^ *PLoS ONE 14:10* (2019) e0212652.

## Data Availability

The original contributions presented in this study are included in the article/Supplementary Materials. Further inquiries can be directed to the corresponding authors.

## Supplementary Materials

The following supporting information can be downloaded at: https://www.mdpi.com/article/10.3390/vetsci13010096/s1, S1: Schematic layout of the Gran Canaria Island Shelter showing the specific location of the animals included in the study (*n* = 20). The number indicates the identification code assigned to each animal; S2: Individual scores for each TEX-Q subscale, reflecting treatment expectations reported by each observer across the three intervention modalities; S3: Associations between contextual variables and behavioral indicators at baseline (mean ± SD; *n* = 20). Significant results (*p* < 0.05) are highlighted in bold.

## Appendix A. The Treatment Expectation Questionnaire (TEX-Q)

Treatment benefit: sum of questions (1) How much relief from anxiety symptoms do you expect to observe with the treatment?, (2) To what extent do you believe the treated animals will benefit from this treatment?, and (3) How much improvement in animal welfare do you expect as a result of the treatment?

Positive impact: sum of questions (4) How significant do you expect the improvement to be in the animals’ welfare and daily behavior?, (5) To what extent do you expect the treatment to improve the animals’ quality of life?, and (6) How much do you expect the treatment to enhance the dogs’ ability to adapt to shelter routines and environmental demands?

Adverse events: sum of questions (7) To what extent do you expect the treatment to involve risks for the dogs?, (8) How much discomfort do you expect the treatment to cause in the treated animals?, and (9) To what extent do you expect the treatment to cause side effects or other undesirable effects in the dogs?

Negative impact: sum of questions (10) To what extent do you expect the treatment to reduce the dogs’ quality of life?, and (11) To what extent do you expect the treatment to limit the dogs’ ability to carry out their daily activities?

Process: sum of questions (12) To what extent do you expect the treatment procedure to be straightforward to administer?, and (13) How satisfied do you expect to be with the procedure or treatment process?

Behavioral control: sum of questions (14) To what extent do you expect to be responsible for the success of the treatment?, and (15) To what extent do you expect your own behavior to influence the success of the treatment?

TEX-Q overall mean score was calculated as mean of all items after inverting items 7–11.

## Appendix B. The Qualitative Behavioral Assessment (QBA) of Shelter Dogs

Negative emotional reactivity: sum of aggressive, fearful, hesitant, and wary.

High emotional reactivity: sum of alert, anxious, attention-seeking, excited, nervous, reactive, and stressed.

Positive emotional reactivity: sum of comfortable, curious, exploratory, interested, playful, relaxed, and sociable.

Low emotional arousal: sum of bored and depressed.
