# Supplementary material for: Evaluation of 1cp-LSD for Enhancing Welfare in Shelter Dogs: A Randomized Blind Trial with Ethological Intervention
Source: Vet Sci. 2026 Jan 19;13(1):96. doi: 10.3390/vetsci13010096 (PMC12846581; doi:10.3390/vetsci13010096)
Supplement: Supplementary file 1 [file vetsci-13-00096-s001.zip › Supplementary file S1.pdf]

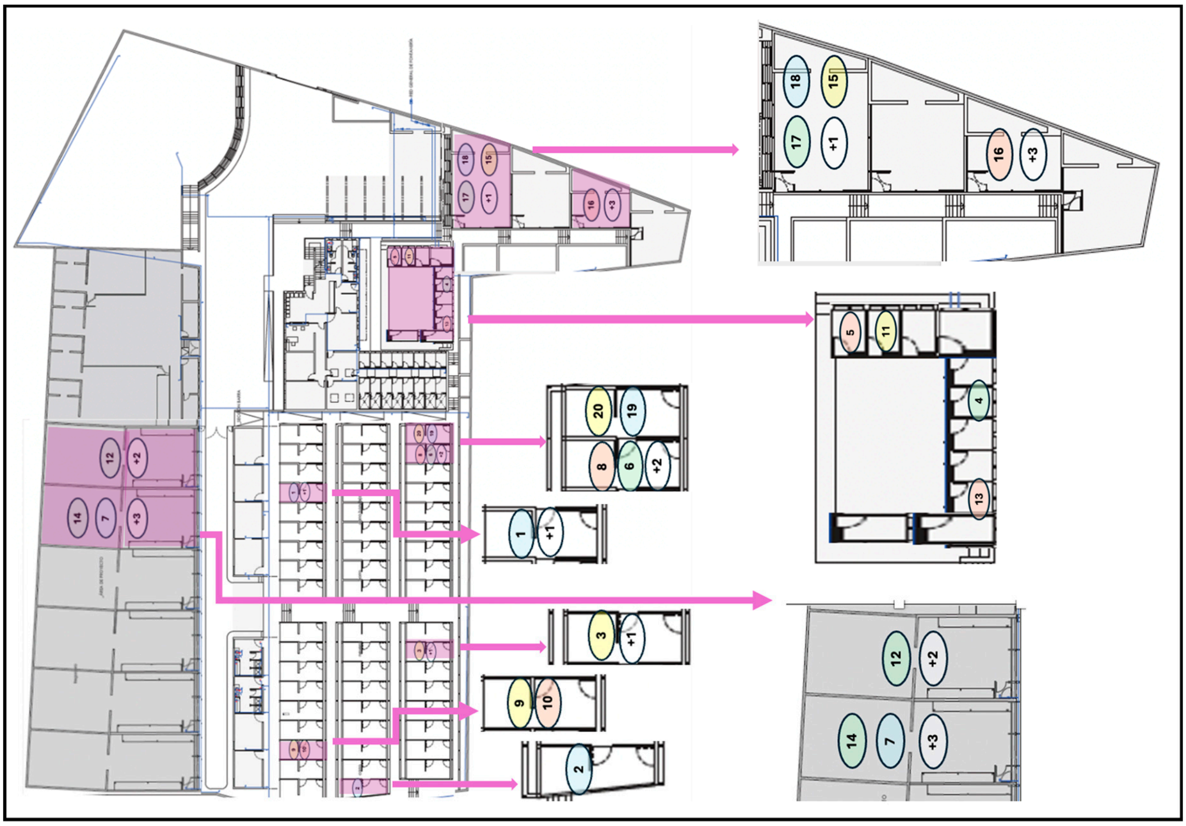

S1. Schematic layout of the Gran Canaria Island Shelter showing the specific location of the animals included in the study (n = 20). The number indicates the identification code assigned to each animal. Orange represents animals in the control group; blue, animals treated with 1cp-LSD; green, those that received the ethology-based intervention; and yellow, those that received both the pharmacological treatment and the ethological intervention. White indicates the number of cohabiting animals not included in the study.
