# Supplementary material for: Evaluation of 1cp-LSD for Enhancing Welfare in Shelter Dogs: A Randomized Blind Trial with Ethological Intervention
Source: Vet Sci. 2026 Jan 19;13(1):96. doi: 10.3390/vetsci13010096 (PMC12846581; doi:10.3390/vetsci13010096)
Supplement: Supplementary file 1 [file vetsci-13-00096-s001.zip › Supplementary file S2.pdf]

Supplementary file S2. Individual scores for each TEX-Q subscale, reflecting treatment expectations reported by each observer across the three intervention modalities.

Intervention modalities and roles*	TEX-Q subscales						
	Treatment benefit	Positive impact	Adverse events	Negative impact	Process	Behavioral control	Overall mean score
Pharmacological treatment							
BO1	6.3	6.3	0.3	0.5	5.0	6.5	7.3
BO2	4.7	3.3	2.3	1.5	5.0	7.5	5.9
NBO1	8.0	6.7	0.0	0.0	3.5	3.0	7.1
NBO2	5.3	5.7	0.7	0.5	5.5	8.0	7.1
Ethology-based intervention							
BO1	6.7	6.3	0.0	0.0	5.0	7.0	7.5
BO2	7.0	7.3	3.0	2.5	3.5	8.5	6.9
NBO1	8.7	8.0	0.0	0.0	7.0	0.0	7.6
NBO2	7.7	8.3	1.3	0.5	7.5	8.0	8.3
Combined treatment							
BO1	8.3	7.3	0.3	0.5	6.5	8.0	8.3
BO2	6.3	4.3	1.3	1.0	7.0	7.5	7.0
NBO1	9.0	7.3	1.0	0.0	7.5	2.5	7.7
NBO2	7.0	6.0	1.7	1.5	7.0	8.5	7.5
P value**	0.178	0.153	0.930	0.972	0.074	0.943	0.167

Abbreviations: TEX-Q, treatment expectation questionnaire; BO, blinded observer; NBO, non-blinded observer.

*Each observer was involved in the following treatment modalities: pharmacological treatment with 1cp-LSD, ethological intervention, and the combined approach. BO1 and BO2 conducted behavioral assessments and delivered the ethological intervention during the experimental phase, remaining blinded to drug allocation. NBO1 and NBO2 were responsible for administering the pharmacological agent and were not involved in the ethological intervention.

**ANOVA test. No significant differences were observed in the pairwise comparisons (Tukey's post hoc test) or in treatment expectancy between blinded and non-blinded observers.
