# Supplementary material for: Evaluation of 1cp-LSD for Enhancing Welfare in Shelter Dogs: A Randomized Blind Trial with Ethological Intervention
Source: Vet Sci. 2026 Jan 19;13(1):96. doi: 10.3390/vetsci13010096 (PMC12846581; doi:10.3390/vetsci13010096)
Supplement: Supplementary file 1 [file vetsci-13-00096-s001.zip › Supplementary file S3.pdf]

Additional file S3. Associations between contextual variables and behavioral indicators at baseline (mean ± SD; n = 20). Significant results (P < 0.05) are highlighted in bold.

	Housing type		Cohoused with other study dogs*		Taken out of enclosure		Behavioral issues with other dogs	
	Group	Individual	No	Yes	No	Yes	No	Yes
Separation anxiety[a]	6.9 ± 1.9	16.7 ± 3.9	7.3 ± 2.7	6.6 ± 3.2	0	10.7 ± 2.3	9.4 ± 3.6	11.8 ± 3.1
P value		**0.030**		0.885		NA		0.626
Stress[b]	3.6 ± 0.3	2.8 ± 0.6	2.4 ± 0.6	4.0 ± 0.2	4.6 ± 0.2	3.0 ± 0.3	3.8 ± 0.2	2.8 ± 0.5
P value		0.230		**0.010**		**0.005**		0.057
Sociability[b]	1.9 ± 0.4	3.2 ±0 .4	3.5 ± 0.6	1.3 ± 0.3	0.6 ± 0.2	2.7 ± 0.3	1.6 ± 0.3	3.1 ± 0.5
P value		0.063		**0.002**		**0.001**		**0.011**
Calmness[b]	1.5 ± 0.3	1.8 ± 1.1	1.8 ± 0.3	1.5 ± 0.4	0.8 ± 0.4	1.9 ± 0.4	1.3 ± 0.3	2.1 ± 0.6
P value		0.826		0.650		0.164		0.199
Fear[b]	3.3 ± 0.4	0.8 ± 0.3	1.5 ± 0.6	4.0 ± 0.6	4.8 ± 0.2	2.0 ± 0.4	3.5 ± 0.4	1.5 ± 0.7
P value		**0.004**		**0.004**		**<0.001**		**0.013**
Excitability[b]	3.4 ± 0.2	3.2 ± 0.7	3.5 ± 0.3	3.3 ± 0.3	3.8 ± 0.5	3.2 ± 0.3	3.5 ± 0.3	3.1 ± 0.4
P value		0.837		0.730		0.256		0.504
Aggressiveness[b]	1.4 ± 0.4	1.8 ± 0.7	0.5 ± 0.3	1.7 ± 0.4	2.6 ± 0.7	1.1 ± 0.3	1.7 ± 0.4	1.1 ± 0.4
P value		0.602		**0.039**		**0.044**		0.353
QBA 1	3.4 ± 0.6	5.0 ± 1.3	5.2 ± 0.7	2.7 ± 0.6	1.4 ± 0.6	4.6 ± 0.5	2.8 ± 0.5	5.2 ± 0.8
P value		0.196		**0.037**		**0.005**		**0.020**
QBA 2	8.1 ± 0.8	5.8 ± 1.4	5.5 ± 0.6	9.1 ± 0.9	11.2 ± 1.2	6.3 ± 0.6	8.7 ± 0.8	5.7 ± 1.0
P value		0.160		**0.037**		**0.001**		**0.034**
QBA 3[c]	12.6 ± 1.0	9.4 ± 1.5	9.2 ± 2.2	13.9 ± 0.9	15.4 ± 0.4	10.7 ± 1.0	13.7 ± 0.8	9.0 ± 1.3
P value		0.115		**0.036**		**<0.001**		**0.005**
QBA 4[c]	22.3 ± 0.5	23.2 ± 4.5	23.0 ± 1.1	22.1 ± 0.6	21.6 ± 1.0	22.8 ± 1.5	22.7 ± 0.6	22.2 ± 2.7
P value		0.737		0.450		0.645		0.874
QBA 5[c]	18.1 ± 1.1	19.6 ± 2.0	22.5 ± 1.8	16.6 ± 1.1	14.4 ± 1.3	19.9 ± 1.0	16.7 ± 1.0	21.2 ± 1.5
P value		0.525		**0.013**		**0.010**		**0.015**

QBA 6[c]	2.8 ± 0.2	5.2 ± 1.3	3.0 ± 0.6	2.7 ± 0.3	2.4 ± 0.4	3.7 ± 0.5	2.9 ± 0.3	4.1 ± 0.9
P value		0.135		0.273		0.175		0.244

Abbreviations: SD, standard deviation; QBA, quality behavior assessment.

*Referred to those dogs who share housing (n = 15).

QBA 1 (emotional regulation and sociability): sum of sociability and calmness; QBA 2 (emotional defensiveness and arousal): sum of fear, excitability, and aggressiveness; QBA 3 (negative emotional reactivity): sum of items 1, 11, 12 and 20 (aggressive, fearful, hesitant, and cautious); QBA 4 (High emotional reactivity): sum of items 2, 3, 4, 9, 14, 16, and 19 (alert, anxious, attention-seeking, excited, nervous, reactive, and stressed); QBA 5 (positive emotional reactivity): sum of items 6, 7, 10, 13, 15, 17 and 18 (comfortable, curious, exploratory, interested, playful, relaxed, and sociable); and QBA 6 (low emotional arousal): sum of items 5 and 8 (bored and depressed).

[a]Journal of Veterinary Behavior (2006) 1, 109-120.

[b]Applied Animal Behaviour Science 213 (2019) 107–116.

[c]X PLoS ONE 14:10 (2019) e0212652

Student's t test was used for the calculation of P values.
